# Importance of micronutrients in bone health of monogastric animals and techniques to improve the bioavailability of micronutrient supplements — A review

**DOI:** 10.5713/ajas.19.0945

**Published:** 2020-03-12

**Authors:** Santi Devi Upadhaya, In Ho Kim

**Affiliations:** 1 Department of Animal Resource and Science, Dankook University, Cheonan 31116, Korea

**Keywords:** Bone Health, Bioavailability, Micronutrient, Minerals, Vitamins

## Abstract

Vitamins and minerals categorized as micronutrients are the essential components of animal feed for maintaining health and improving immunity. Micronutrients are important bioactive molecules and cofactors of enzymes as well. Besides being cofactors for enzymes, some vitamins such as the fat-soluble vitamins, vitamin A and D have been shown to exhibit hormone-like functions. Although they are required in small amount, they play an influential role in the proper functioning of a number of enzymes which are involved in many metabolic, biochemical and physiological processes that contribute to growth, production and health. Micronutrients can potentially have a positive impact on bone health, preventing bone loss and fractures, decreasing bone resorption and increasing bone formation. Thus, micronutrients must be provided to livestock in optimal concentrations and according to requirements that change during the rapid growth and development of the animal and the production cycle. The supply of nutrients to the animal body not only depends on the amount of the nutrient in a food, but also on its bioavailability. The bioavailability of these micronutrients is affected by several factors. Therefore, several technologies such as nanoparticle, encapsulation, and chelation have been developed to improve the bioavailability of micronutrients associated with bone health. The intention of this review is to provide an updated overview of the importance of micronutrients on bone health and methods applied to improve their bioavailability.

## INTRODUCTION

Micronutrients are essential elements required by organisms in small quantities for normal body function e.g. vitamin and minerals. Vitamins are organic micronutrients that are necessary in minute amounts for the normal function of many metabolic pathways, gene regulation and immune functions [1]. Vitamins play an influential role in the regulation of body functions, promoting resistance to diseases thereby keeping the body healthy. The deficiency of a vitamin can lead to disease or death. Vitamins are classified as fat-soluble and water-soluble. Fat-soluble vitamins including vitamins A, D, E, and K are vitamins stored in the adipose tissue and released as per the need of the body. B vitamins (thiamine, riboflavin, pyridoxine, pantothenic acid, niacin, folic acid, choline and vitamin B_12_) and vitamin C are water-soluble vitamins. As water passes through the body, it carries out water-soluble vitamins. Thus, these vitamins need to be consumed every day by farm animals. However, vitamin C can be synthesized in tissues by animals under normal conditions [1] but under different stress conditions, it should be provided to the animals. Unlike monogastric animals, water-soluble B vitamins are made by microorganisms in the rumen of a ruminant animal [2].

Minerals are inorganic nutrients that a re required in small quantities but participate in orchestration of different biological processes that drive normal growth, development, and function. Minerals are also essential for the formation of bones and teeth; as essential constituents of body fluids and tissues; as components of enzyme systems and for normal nerve function. Minerals are classified as macrominerals or microminerals. Macrominerals including calcium, chlorine, magnesium, phosphorus, potassium, sodium, and sulfur are needed in relatively higher amount (g/kg body weight dimension) than microminerals or trace minerals such as chromium, cobalt, copper, fluorine, iodine, iron, manganese, molybdenum, selenium, zinc, silicon and boron. It is well-established that the deficiency or inadequate amount of minerals in the diet may impair productivity, immune functions and health [3,4].

Considering the massive changes in growth potential and management in commercial pig and poultry production over the coming decades and how these may influence the vitamin nutrition of animals, a new concept of optimum vitamin nutrition (OVN) has become essential. Thus, various sources have recommended vitamin doses so as to meet the needs of body in poultry (Tables 1, 2) and pigs as shown in (Tables 3, 4). An appropriate and specific supply during the specific physiological phases of animals leads to positive results that go beyond the initial objective of preventing deficiency. Thus, with the rapid growth and development of animals and their production cycle, the supplementation of micronutrients must be adjusted according to their requirements to meet their body needs as a means of enhancing growth and reproductive performance, improving immunity indices, maximize mineral digestibility, improve bone health and egg shell quality.

## MICRONUTRIENTS IN BONE HEALTH

Bony skeleton is an important organ that not only provides mobility, support and protection to the body but also acts as a reservoir of major essential minerals such as Ca and P. The growth of the skeleton, its response to mechanical forces, and its role as a mineral storehouse are all dependent on the proper functioning of a number of circulating hormones that are responsive to changes in blood calcium, phosphorus as well as other minerals. Genetic abnormalities, nutritional deficiencies, hormonal disorders, lack of exercise, immobilization can result in the formation of weak, poorly mineralized bone having negative effects on bone mass and strength leading to osteoporosis and osteomalacia [5].

An important modifiable factor in the prevention of os teoporosis (disease in which the density and quality of bone are reduced) as well as in bone development and maintenance of bone mass is nutrition [6]. Micronutrients and macronutrients can influence bone health [7,8]. Clinical trials showed micronutrients can potentially have a positive impact on bone health, preventing bone loss and fractures, decreasing bone resorption and increasing bone formation [9]. In addition to their passive roles as substrate for bone formation, dietary calcium and protein play active roles in bone metabolism, other vitamins and minerals are also needed for metabolic processes related to bone, directly or indirectly. Micronutrients involved in bone health are as follows:

### Calcium and phosphorous

The most abundant cation in the body is calcium. About 99% of calcium (Ca) exists in the mineral phase of bone and the remaining 1% is present within the extracellular and intracellular fluids [6]. Likewise, about 85% of body phosphorous (P) in phosphate form is present in bone. Hormonal and physicochemical factors stimulate Ca and phosphate to interact in many fundamental processes in the body and Ca and P are complementary for bone growth and development [10]. For sustaining mineral homeostasis and bone metabolism, the ratio of Ca to P is critical [11]. Lagos et al [12] determined that if P is provided in the required level of 0.33% in pigs, the optimum ratio of Ca:P is 1.39:1 or 1.25:1 to maximize daily gain and gain: feed respectively whereas for the optimization of bone ash, the ratio is 1.66:1. The optimal levels of dietary standardized total tract digestible calcium for retention of calcium and phosphorus were suggested at or above 0.60% and 0.49% respectively [12]. The bone formation requirements that have a close connection with the health and welfare of the animals determine the need for Ca and P. For a proper functional musculoskeletal system, growth and performance traits should be combined with adequate bone formation [13]. Thus, the surpluses or deficiency of Ca and P have remarkable effects on bone mineral content and density, bone development and bone composition. The Ca and P dependent processes depend on the uptake, excretion and availability of these elements. Therefore, considerable attention has been paid to the regulation of plasma parathyroid hormone, calcium-binding protein, calcitriol/1, 25(OH)_2_D_3_ [14,15] and insulin-like growth factor-1 concentration [16]. Ca and P play a central role not only in the formation of bone during the growth of piglets, these minerals and associated endocrine factors are required for the development of soft tissues. In a pig study, Heyer et al [17] suggested that there exist links between dietary P and the immune system, indicating that the mineral nutrient is equally important for improved immune functions. A strong linkage of bone and immune system development is facilitated by regulatory processes in the bone marrow that control immune functions and hematopoiesis [18].

### Magnesium

Magnesium is an essential cofactor in 80% of all cellular enzymes and is an important contributor to bone health. About 60% of the body magnesium is found in bone and it makes up about 1% of the total bone mineral content [19]. It is necessary for the conversion of vitamin D into its active form [20]. The enzyme that is required for forming new calcium crystals, alkaline phosphatase, also requires magnesium for activation, and if its levels are low, abnormal bone crystal formation can result [21]. Even mild magnesium deficiency is reported to be a leading risk factor for osteoporosis [22]. Research has been done in recent years that shows osteoporosis can be a direct result of calcium that hasn’t been metabolized by the body due to a lack of magnesium.

### Zinc

Zinc is an important element in bone metabolism which is needed to produce the matrix of collagen protein threads upon which the bone-forming calcium–phosphorus compound is deposited. It acts as a co-factor in over 200 enzymatic reactions and plays a crucial role in bone health. Zinc also stimulates the production of enzymes responsible for the degradation and recycling of worn-out bits of bone protein. Zn may impact the normal physiological action of vitamin D on calcium metabolism and interfere with the anabolic activity of vitamin D on bone tissue [23,24].

### Copper

Copper is involved in many biological processes including enzymatic reactions and bone health. The formation of bone, mineralization of skeleton, and the integrity of the connective tissue are significantly influenced by copper [25,26]. Copper deficiency may result in impaired bone and cholesterol metabolism [27]. For the cross-linking of collagen fibrils, lysyl oxidase, a copper-containing enzyme is essential as it helps to increase the mechanical strength of the protein to form strong, flexible connective tissue [28]. Copper supplementation to rats suffering an ovariectomy-induced reduction of bone mass had a positive effect suggesting its therapeutic potential in osteoporosis [29].

### Silicon and boron

Several studies demonstrated that silicon is also an essential trace mineral for normal bone development, cartilage growth, and improved bone quality [30,31]. Some evidence indicates the involvement of silicon with calcium metabolism and the formation and stabilization of the extracellular bone matrix [32,33]. It can regulate calcium turnover that influences the processes of bone calcification and decalcification [34]. Synergistic effects of calcium and silicon for the stimulation of bone formation have been reported by Maehira et al [35]. Incharoen et al [31] have also suggested that the supplementation of silicon into the diet as a mineral additive enhanced the bone and meat quality in broilers. The ability of silicon to reduce lameness in broilers has been detected in the studies by Short et al [36] and Nakhon et al [37].

Boron is increasingly recognized as an element that has an influence on bone health by stabilizing and extending the half-life of vitamin D and estrogen [38–40]. The supplementation of boron in the form of boric acid has been shown to increase bone strength in rats and laying hens [41,42]. In boron deficient rats, it was found that healing of the alveolar bone, a ridge of compact bone that contains the tooth sockets on the maxillae and mandible was inhibited indicating that boron also influences bone health.

### Vitamins

Among vitamins, vitamin D is quite unique since it can be obtained from the diet as well as synthesized in the body from exposure to ultraviolet B radiation from sunlight. The form of vitamin D used in supplement products can be either vitamin D_2_ (ergocalciferol) or vitamin D_3_ (cholecalciferol). Vitamin D_3_ is also synthesized in the skin from 7-dehydrocholesterol under the influence of ultraviolet B radiation from sunlight through a two-step process in which the B ring is broken by UV light radiation forming pre-D_3_ that isomerizes to D_3_ in a thermo-sensitive process [43]. Endogenous synthesis, however, is considered the most available source of vitamin D [44]. Compared to what is typically consumed in the diet, higher amounts of vitamin D can be obtained from sun exposure. However, in intensive farming systems, animals are kept in close confinement and may not have enough exposure to sunlight leading to inadequate vitamin D synthesis. A deficiency in young pigs results in rickets, stiffness and lameness, enlargement of the joints and general unthriftiness. In mature and market animals, bone fractures are common if vitamin D is deficient. Thus, supplemental vitamin D is needed for the efficient absorption and metabolism of calcium and phosphorus and therefore is required for normal calcification of bones [45]. The secretion of parathyroid hormone is regulated by vitamin D thereby stimulating several tissues with vitamin D receptors [46]. Regardless of origin, vitamin D is an inactive prohormone and must first be metabolized to its hormonal form before it can function. Thus, to generate the biologically active form, vitamin D needs to be processed further. This activation process occurs in 2 steps: i) within the liver, cholecalciferol is hydroxylated to 25-hydroxycholecalciferol (25[OH] 2D) by the enzyme 25-hydroxylase; and ii) within the kidney, 25-hydroxycholecalciferol is converted to 1, 25(OH)_2_D by the enzyme 1α hydroxylase [47,48]. In human studies, it was demonstrated that each microgram of orally consumed 25-hydroxyvitamin D_3_ was 4.2 to 5 times more potent in raising serum 25(OH)D in older adults in winter than was an equivalent amount of vitamin D_3_ [44]. It has been reported that 25(OH) D_3_ is more active than vitamin D_3_ and allows more efficient utilization [49]. However, Garcia and coworkers [50], noted that the different metabolites D_3_, 25(OH) D_3_, 1, 25(OH)_2_ D_3_, and 1α(OH)D_3_ used showed similar results for bone parameters in broiler chickens. A study by Amundson [51] indicated that sows femur’s mechanical properties were improved with the inclusion of vitamin D to the diet. Besides bone health, vitamin D also has an influential role in the regulation of myogenic genes [52]. However, excessive amounts of vitamin D in the feed or as an injectable are harmful due to deposits of calcium in soft tissues [53].

The association between various B vitamins (B _2_, B_6_, folate, or B_12_) and a lower risk of osteoporosis are inconsistent among studies or across different B vitamins. For instance, B_6_ deficient rats had osteoporotic veins with cavities and less new bones [54] and vitamin B_6_ deficient chicks had decreased cortical thickness, osteoid in trabecular bone, reduced secondary ossification centers and coarse trabeculation [55]. However, no difference in bone strength, bone area was observed in vitamin B_12_-deficient rats [56] and no significant difference in osteocalcin, callus stiffness and size was seen in folate and vitamin B-_12_ deficient mice [57].

Vitamin K activates a protein called osteocalcin, which builds and heals bone [58]. Transcription and translation of osteocalcin gene are regulated by 1, 25(OH)_2_ D_3_ [59], but its ability to bind to calcium ions depends on the vitamin K [60]. In a review, Akbari and Rasouli-Ghahroudi [61] suggested that adding vitamin K as an adjunct to the bone materials may stimulate bone cells and their progenitors to produce native bone with promising results reflecting the importance of vitamin K in bone health.

## BIOAVAILABILITY OF MICRONUTRIENTS (VITAMINS AND MINERALS)

Regardless of whether micronutrients are consumed in the form of feed or supplements, it is believed that not 100% of the ingested nutrients will be absorbed because of bioavailability issues. Understanding nutrient bioavailability helps to optimize diets and set appropriate nutrient recommendations. The term bioavailability is used to refer to the sequential metabolic events of nutrient utilization, and it includes digestion, absorption, enzymatic transformation, and excretion [62]. To make the nutrient bioavailable, it should be bio-accessible [63]. Nutrients can be made bio-accessible by the processes of mastication and enzymatic digestion of the food in the mouth, followed by swallowing and mixing with acid and enzymes in the gastric juice, and absorption after finally being released into the small intestine, which is the major site of nutrient absorption [64]. Thus, first step is to liberate nutrients from food matrix and covert them to chemical forms which can then easily bind to and enter the gut cells.

Essential nutrients should be provided in appropriate amounts and in the forms that are biologically utilizable for the efficient production of livestock and poultry and the maintenance of their normal life functions [65–67]. Deficiencies of certain nutrients occur frequently in diets consisting of common feed ingredients, and these nutrients must be provided in a supplemental form. Degree of bioavailability influences not only dietary requirements but also tolerance of a nutrient. Thus, it is important to know the bioavailability of nutrients in both common feed ingredients and dietary supplements that may be used in animal feeding.

Numerous factors that influence the bioavailability of nu trients are the chemical form of nutrient; the nutrient matrix; the nutrient absorption enhancers and inhibitors; metabolic ability after absorption; state of health, genetic factors, age of the host; as well as other individual factors [68].

For instance, the bioavailability of lipid-soluble nutrients is significantly influenced by their physicochemical availability so as to be incorporated into mixed micelles during digestion. Upon ingestion and initial digestion within the stomach, there is a possibility of release of lipid-soluble components. The factors influencing the release include localization within the food matrix; physical break-up of the food; breakdown of the food particles through chemical and enzymatic reactions; and the presence of a suitable lipid phase in the form of emulsion droplets or free lipid [69].

The occurrence of competition for common binding sites or carriers has been reported in minerals having similar chemical properties. For example, iron, zinc, and copper are typical examples of competitive inhibitors [70]. Besides this, the nutritional status of host for specific micronutrients, notably iron, has a major effect on absorption. For instance, the status of vitamin A can decrease iron bioavailability by preventing hemoglobin formation [71]. In addition, the bioavailability of Fe is impaired due to the chelation of Fe with dietary polyphenols in the lumen of the intestine. Fe bioavailability can be enhanced by ascorbate which reduces ferric iron to the ferrous form of iron which is less reactive with phytate or polyphenols [72].

A number of host-related physiological factors influence the absorption and utilization of nutrients. Most of these factors are key participants in the body’s homeostatic regulatory mechanisms such as nutritional status, stage of development, gastric and intestinal secretions, mucosal cell regulation, and gut microflora. The role of gastrointestinal (GI) factors is clearly seen in the absorptive pathway of vitamin B_12_. This vitamin requires gastric acid to be released from the gastric mucosa which then undergoes a sequence of binding to cobalophilin, release from cobalophilin, binding to the protein “intrinsic factor (IF)” and finally absorption of the intact IF-vitamin B_12_ complex in the lower intestine [73]. The functional impairment of the gastric mucosa can compromise the production of IF and cobalophilin eventually inhibiting vitamin B_12_ bioavailability.

### Technologies for the improvement of supplemental micronutrient bioavailability

#### Nano engineering and encapsulation/coating

Nano engineering ensures the development of materials with novel properties having wider range of applications and can be prepared from both organic and inorganic materials [74,75]. Nano-engineering of materials enhances their bioavailability by protecting them against chemical conditions in the GI tract, and allowing the controlled release within the GI tract or by an improved transfer through the intestinal wall [76,77]. The characteristics of nano-engineered materials affecting the final nutritional value include particle size, physical state of nano-materials and their surface properties [78].

Several encapsulated nanoparticles have been designed and tested for their potential use as delivery systems for vitamin D and vitamin E so as to optimize supplementation strategies thereby improving the health benefits via encapsulation, protection and/or controlled/sustained release [79–83]. Although ZnO and Cu salts in high doses as supplements to piglets’ diet, stimulate piglets’ daily gain and decrease feed conversion factor, the application of high concentrations of these metal additives could lead to an adverse effect on environment and public health hazard. In the meantime, ZnO applied at doses 2,500 to 3,000 mg/kg feed can contribute to the development of antimicrobial resistance and may modify piglets’ immune response via the regulation and expression of related genes [83]. The application of nano sized ZnO/Cu particles could notably reduce the dietary inclusion rate and environmental pollution while preserving the beneficial impact on pigs’ health [84]. The degree of the reduction of piglet diarrhea incidence observed with low dose ZnO nanoparticles (600 mg Zn/kg) supplemented to the basal diet of weaning piglets as compared with that of high dose of traditional ZnO (2,000 mg Zn/kg) has been suggested to be mediated by improving intestinal microbiota and inflammation response in piglets, and could contribute in reducing zinc environmental pollution [84]. Our study also demonstrated that lipid-matrix coated ZnO at a low dose could substitute for the conventional high dose ZnO in weaning pigs [85]. Vahjen et al [86] demonstrated that the new form of modified ZnO led to a reduction in bacterial growth in the GI tract more rapidly than analytical grade ZnO which was consistent with our study which demonstrated that the dietary supplementation with modified ZnO increased growth rate, bioavailability and reduced fecal scores in weanling pig [87].

#### Chelation

Chelation is the process in which organic molecules such as amino acids or peptides trap or encapsulate certain metal ions like Ca, Mg, Fe, Co, Cu, Zn, and Mn. Several bonds with a single metal ion can be formed with these organic molecules. The chelate creates compatibility between the two charges by encapsulating the positively charged nutrient and neutralizing it. The nutrient is then able to move freely into the animal body. Chelation allows nutrients to be absorbed by animals with ease if there is lack of competitive antagonism among minerals at the site of absorption. The bioavailability of mineral supplements ingested as mineral salts has been observed in animal studies [88,89]. Generally, the best sources of supplemental elements include sulfates, chlorides, phosphates, acetates, citrates and gluconates or carbonates of Ca and Zn whereas sulfides and oxides are generally poor sources of mineral elements [90]. Amino-acid chelates are reported to have significantly higher absorption rates in the intestine compared with soluble inorganic metal salts [91]. Jiao et al [92] demonstrated that increasing dose of zinc aspartic acid chelate (0%, 0.1%, 0.2%, 0.3%) supplementation imparted beneficial effects on the performance and digestibility of nutrients in growing pigs. However, reported results regarding the bioavailability of Zn chelates and traditional inorganic forms are conflicting [93]. Guo and coworkers [94] demonstrated the enhancement of Ca absorption and significant increase in femur bone mineral density and femur Ca content in rats when Ca was supplemented in the form of Ca alginate nanoparticles loaded with collagen peptide chelated Ca suggesting that they could prevent Ca deficiency. In addition, Chen et al [95] demonstrated that a calcium-chelating peptide complex prepared from tilapia skins could promote bone formation and an increase in bone collagen in mice via a better bioavailability of Ca. Zinc in the form of methionine hydroxy analog chelate zinc has been found to be more stable than other organic Zn forms and has higher bioavailability in broilers [96] and laying hens from 39 to 52 weeks of age [97]. In a trial with 120,000 broilers, Manangi et al [98] reported that supplementing the birds with reduced level of Zn (32 ppm), copper (8 ppm), and manganese (32 ppm) as methionine hydroxy analogue chelates significantly improved footpads and reduced trace mineral excretion compared to birds fed higher levels of Zn (100 ppm), Cu (125 ppm), and Mn (90 ppm) suggesting that chelating technology enhances the bioavailability of nutrients.

## CONCLUSION

The importance of micronutrients, for example, vitamins and minerals, in sufficient amounts needed for bone health of humans and animals is indisputable. Therefore, different technologies are currently being employed to improve the bioavailability of these micronutrients so as to ensure the optimal supplementation of these micronutrients for better health status including bone health and with an additional benefit of reducing their impact on the environment.

## Figures and Tables

**Table 1:** Recommended vitamin levels for broiler starter to finisher period by different sources

Item	Unit	Starter-grower					Finisher			
	
		NRC (1994)	INRA (1984)	FEDNA (2008)	DSM OVN (2011)		NRC (1994)	INRA (1984)	FEDNA (2008)	DSM OVN (2011)

				0–18 days	Starter	Grower				
Vitamin A	IU/kg	1,500	10,000	9,000–15,000	11,000–15,000	10,000–125,000	1,500	10,000	7,000–9,000	10,000–12,500
Vitamin D_3_	IU/kg	200	1,500	3,000–3,800	3,000–5,000	3,000–5,000	200	1,500	2,000–2,800	3,000–5,000
25-OH-D_3_ (HyD)	mg/kg	-	-	-	0.069	0.069	-	-	-	0.069
Vitamin E	IU/kg	10	15	20–45	150–300	50–100	10	10	20–25	50–100*
Vitamin K	mg/kg	0.5	5	2.0–3.1	3.0–4.0	3.0–4.0	0.5	4	1.7–2.2	3.0–4.0
Vitamin B_1_	mg/kg	1.8	0.5	1.0–2.5	3.0–4.0	2.0–3.0	1.8		0.3–1.3	2.0–3.0
Vitamin B_2_	mg/kg	3.6	4	5.0–7.5	8.0–10.0	7.0–9.0	3.6	4	3.0–5.5	6.0–8.0
Vitamin B_6_	mg/kg	3.5	-	2.5–4.0	4.0–6.0	4.0–6.0	3.5	-	0.6–2.4	4.0–6.0
Vitamin B_12_	mg/kg	0.01	0.01	0.016–0.022	0.02–0.04	0.02–0.03	0.01	0.01	0.008–0.015	0.02–0.03
Niacin	mg/kg	35	25	40–65	60–80	60–80	30	15	20–35	50–80
Panthothenic acid	mg/kg	10	5	10.0–16.0	15.0–20.0	12.0–18.0	10	5	8.0–10.0	10.0–15.0
Folic acid	mg/kg	0.55	0.2	1.0–1.5	2.0–2.5	2.0–2.5	0.55	-	0.3–0.7	2.0–2.5
Biotin	mg/kg	0.15		0.09–0.18	0.2–0.4	0.2–0.3	0.15	-	0.02–0.095	0.2–0.3
Vitamin C	mg/kg	-	-	-	100–200**	100–200**	-	-	-	100–200**
Choline	mg/kg	1,300	500	300–500	400–700	400–700	1,000	500	175–250	400–600

NRC, National Research Council; INRA, Institut National de Recherche Agronomique (National Institute for Agricultural Research); FEDNA, Fundación Española para el Desarrollo de la Nutrición Animal (Foundation for the Development of Animal Nutrition); OVN, optimum vitamin nutrition.

* Additional 150 mg/kg for meat quality.

** Recommended under heat stress.

Adapted from DSM Nutritional Product Limited 2012, 5m Publication, UK [99].

**Table 2:** Recommended vitamin levels for laying hens by genetic companies and different sources

Item	Unit	ISA brown (2010)	Hy-line (2010)	Lohman (2009)	DSM (OVN) (2011)	FEDNA (2008)	Maximum level in literature	Responses
Vitamin A	IU/kg	10,000	8,800	10,000	8,000–12,000	8,000–10,000	12,000	Better immune response in heat stress [100]
Vitamin D_3_	IU/kg	2,500	3,300	2,500	3,000–4,000	2,000–3,000	6,000/15,000	Improved bone strength in laying hens [101]
25-OH-D_3_ (HyD)	mg/kg	-	0.055	-	0.069	-	0.069	Improved egg production [102]
Vitamin E	IU/kg	20	16.5	10.0–30.0	20–30	8.0–20	500	Reduced chronic stress [103]
Vitamin K	mg/kg	3.0	2.2	3.0	2.5–3.0	1.4–2.1	10	Improved bone strength in layers and pullets [104]
Vitamin B_2_	mg/kg	5.0	5.5	4.0	5.0–7.0	4.0–6.0	8.8	Enhanced production [105]
Vitamin B_6_	mg/kg	5.0	3.3	3.0	3.5–5	1.5–3.0	6.0	Prevent bone deformities[106]
Vitamin B_12_	mg/kg	0.015	0.022	0.015	0.015–0.025	0.009–0.015	0.036	
Niacin	mg/kg	40	28	30	30–50	18–35	250–1,550	Enhanced feed efficiency, improved shell quality and reduced yolk cholesterol [107]
Panthothe-nic acid	mg/kg	12	6.5	8.0	8.0–12.0	7.0–10.0	-	
Folic acid	mg/kg	0.75	0.6	0.5	1.0–1.5	0.2–0.6	1.5	Performance improvement in old hens [108]
Biotin	mg/kg	0.05	0.055	0.025	0.1–0.15	0.035–0.08	-	
Vitamin C	mg/kg	100	-	-	100–200*	-	2,000	Improved bone shell and mineralization [109]
Choline	mg/kg	1,400	110	400	300–500	150–250	1,500–2,000	Reduced fat deposition in liver [110]

OVN, optimum vitamin nutrition; FEDNA, Fundación Española para el Desarrollo de la Nutrición Animal (Spanish Foundation for the Development of Animal Nutrition).

* Recommended under heat stress condition.

Adapted from DSM Nutritional Product Limited [99].

**Table 3:** Recommended vitamin levels for weaner-finisher pigs by various sources

Item	Unit	NRC (2012)							DanBred			DSM (OVM, 2011)			
		
		Piglets (kg)			Growing-Finisher (kg)				Weaner-Finisher (kg)			Weaner-Finisher (kg)			
			
		5–7	7–11	11–25	25–50	50–75	75–100	100–135	<9	9.0–30	30–100	<5	5.0–30	30–70	>70
Vitamin A	IU/kg	2,200	2,200	1,750	1,300	1,300	1,300	1,300	8,000	5,000	4,000	10,000–20,000	10,000–15,000	7,000–10,000	5,000–8,000
Vitamin D_3_	IU/kg	220	220	200	150	150	150	150	800	500	400	1,800–2,000	1,800–2,000	1,500–2,000	1,000–1,500
25-OH-D_3_ (HyD)	mg/kg	-	-	-	-	-	-	-	-	-	-	0.05	0.05	0.05	0.05
Vitamin E	IU/kg	16	16	11	11	11	11	11	130	130	36	100–150	100–150	60–100	60–100
Vitamin K	mg/kg	0.5	0.5	0.5	0.5	0.5	0.5	0.5	2.0	2.0	2.0	8.0–10	5.0–6.0	2.0–4.0	2.0–4.0
Vitamin B_1_	mg/kg	1.5	1.0	1.0	1.0	1.0	1.0	1.0	2.0	2.0	2.0	3.5–5.5	3.0–5.0	2.0–3.0	1.0–2.0
Vitamin B_2_	mg/kg	4.0	3.5	3.0	2.5	2.0	2.0	2.0	4.0	4.0	2.0	10.0–15.0	10.0–15.0	7.0–10.0	6.0–10.0
Vitamin B_6_	mg/kg	7.0	7.0	3.0	1.0	1.0	1.0	1.0	3.0	3.0	3.0	6.0–8.0	6.0–8.0	2.5–4.5	2.0–3.5
Vitamin B_12_	mg/kg	0.02	0.017	0.015	0.01	0.005	0.005	0.005	0.02	0.02	0.02	0.05–0.07	0.04–0.06	0.03–0.05	0.03–0.05
Niacin	mg/kg	30	30	30	30	30	30	30	20	20	20	60–80	33–55	20–40	20–40
Panthothenic acid	mg/kg	12	10	9.0	8.0	7.0	7.0	7.0	10	10	10	30–50	25–45	25–45	25–45
Folic acid	mg/kg	0.3	0.3	0.3	0.3	0.3	0.3	0.3	-	-	-	1.5–3.0	1.5–2.5	1.0–1.5	0.5–1.0
Biotin	mg/kg	0.08	0.05	0.05	0.05	0.05	0.05	0.05	0.02	0.02	0.1	0.20–0.04	0.2–0.4	0.15–0.30	0.10–0.20
Vitamin C	mg/kg	-	-	-	-	-	-	-	-	-	-	100–200	100–200	0	0
Choline	mg/kg	600	500	400	300	300	300	300	-	-	-	500–800	250–400	150–300	100–200

NRC, National Research Council; OVN, optimum vitamin nutrition.

Adapted from DSM Nutritional Product Limited [99] and National Research Council [111].

**Table 4:** Recommended dietary vitamin levels for breeder pigs by various sources

Item	Unit	NRC (2012)			DanBred		DSM (OVN, 2011)		
		
		Gestation	Lactation	Boars	Gestation	Lactation	Gilts	Sows	Boars
Vitamin A	IU/kg	4,000	2,000	4,000	8,000	8,000	10,000–12,500	10,000–15,000	10,000–15,000
Vitamin D_3_	IU/kg	800	800	200	800	800	1,800–2,000	1,500–2,000	1,500–2,000
25-OH-D_3_ (HyD)	mg/kg	-	-	-	-	-	0.05	0.05	0.05
Vitamin E	IU/kg	44	44	44	36	150	80–100	100–150	100–150
Vitamin K	mg/kg	0.5	0.5	0.5	2.0	2.0	1.5–3	4.5–5	4.5–5
Vitamin B_1_	mg/kg	1.0	1.0	1.0	2.0	2.0	1.0–2.0	2.0–2.5	1.0–2.0
Vitamin B_2_	mg/kg	3.75	3.75	3.75	5.0	5.0	6.0–10.0	6.0–10.0	6.0–10.0
Vitamin B_6_	mg/kg	1.0	1.0	1.0	3.0	3.0	3.5–5.5	3.5–5.5	3.5–5.5
Vitamin B_12_	mg/kg	0.015	0.015	0.015	0.02	0.02	0.03–0.05	0.03–0.05	0.03–0.05
Niacin	mg/kg	10	10	10	20	20	20.0–30.0	25–45	25–45
Panthothenic acid	mg/kg	12	12	12	15	15	15–30	30–35	20–30
Folic acid	mg/kg	1.3	1.3	1.3	1.1	1.1	3.5–5.5	3.5–5.5	3.5–5.5
Biotin	mg/kg	0.2	0.2	0.2	0.02	0.02	0.30–0.50	0.50–0.80	0.50–0.80
Vitamin C	mg/kg	-	-	-	-	-	200–300	200–300	200–500
Choline	mg/kg	1,250	1,000	1,250	-	-	325–500	500–800	500–800

NRC, National Research Council; OVN, optimum vitamin nutrition.

Adapted from DSM Nutritional Product Limited [99] and National Research Council [111].
